# Supplementary material for: Genomic Characteristics and Molecular Epidemiology of Multidrug-Resistant Klebsiella pneumoniae Strains Carried by Wild Birds
Source: Microbiol Spectr. 2023 Feb 22;11(2):e02691-22. doi: 10.1128/spectrum.02691-22 (PMC10101063; doi:10.1128/spectrum.02691-22)
Supplement: Supplemental file 1 — Supplemental material. Download spectrum.02691-22-s0001.pdf, PDF file, 0.4 MB [file spectrum.02691-22-s0001.pdf]

Supplementary materials

Genomic characteristics and molecular epidemiology of multidrug resistant *Klebsiella pneumoniae* carried by wild birds

Authors: Xue Wang[1*], Jianan Zhao[1*], Fang Ji[1], Meng Wang[1,2], Bin Wu[1], Jianhua Qin[2], Guoying Dong[3], Ruili Zhao[4], Chengmin Wang[1#]

Affiliations:

1. Guangdong Key Laboratory of Animal Conservation and Resource Utilization, Institute of Zoology, Guangdong Academy of Science, Guangzhou 510260, Guangdong Province, China
2. College of Veterinary Medicine, Agricultural University of Hebei, Baoding 071001, Hebei Province, China
3. College of Global Change and Earth System Science, Beijing Normal University, Beijing 100875, China.
4. College of Animal Science and Veterinary Medicine, Tianjin Agricultural University, Tianjin, China.

[*]Equally to this work.

[#]Corresponding author. E-mail: wangchm@giz.gd.cn

Figure S1. Homologous structure comparison of similar plasmids pM911-1.1, pS90-2.3 based on software Mauve for multi-drug resistant plasmids.

A

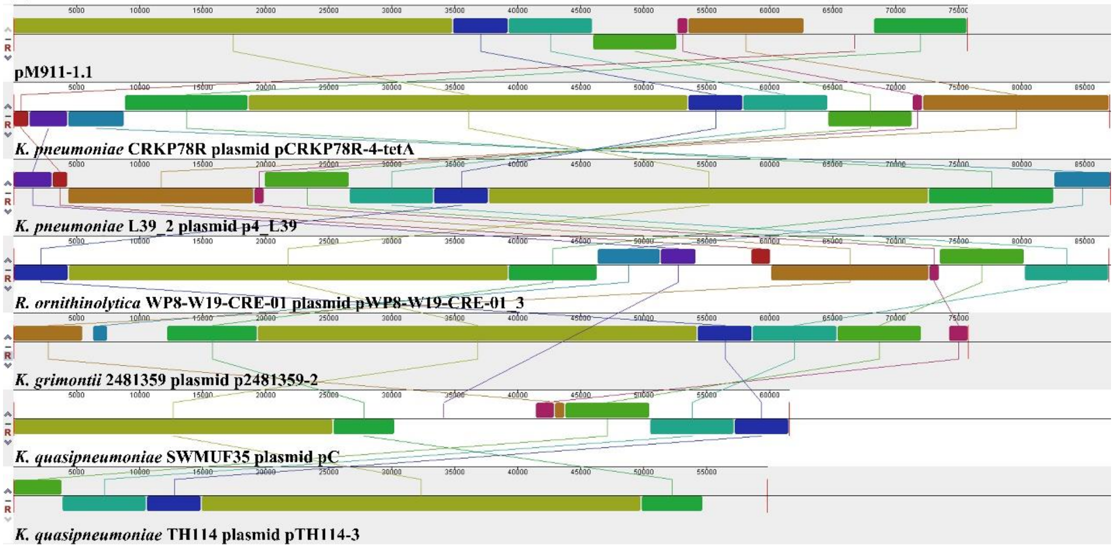

B

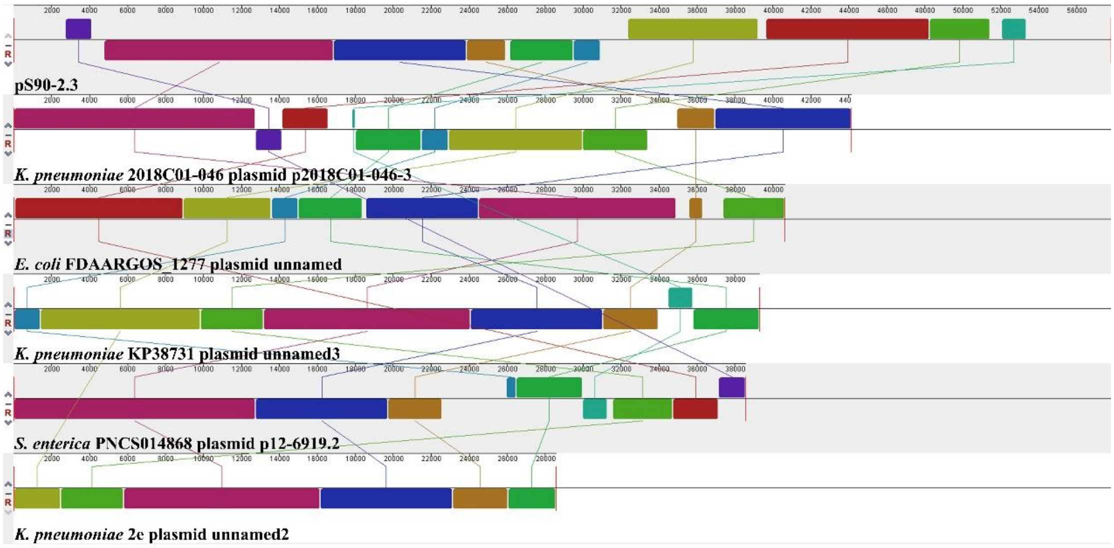

Table S1. Information of strains

Strain name	Strain accession	Host	Genome size of strain(kb)	Plasmid name	Plasmid accession	Plasmid size of strain(kb)	Number of drug resistance genes	Number of gene islands	Number of prophage
S90-2	CP063881.1	Chuckar	5374.786	-	-	-	30	12	0
				pS90-2.1	CP063882.1	110.388	0	1	0
				pS90-2.2	CP063883.1	109.675	0	1	1
				pS90-2.3	CP063884.1	57.825	9	2	1
S141	CP063871.1	Red-breasted parakeet	5383.698	-	-	-	27	9	0
				pS141.1	CP063872.1	194.302	1	5	0
				pS141.2	CP063873.1	112.160	0	1	0
M911-1	CP064129.1	Sun parakeet	5211.192	-	-	-	27	15	0
				pM911-1.1	CP064130.1	75.711	3	2	0
				pM911-1.2	CP064131.1	85.824	0	1	1
				pM911-1.3	CP064132.1	21.377	0	0	1
S130-1	CP063865.1	Black-collared starling	5249.027	-	-	-	27	10	0
				pS130-1	CP063866.1	150.355	1	3	0

Table S2. Source of homologous gene fragment in MDR region of drug resistant plasmid

MDR region of drug-resistant plasmid in this study	Name	Type	Accession	Strain	Host	Date	Country
pM911-1_MDR	pCRKP78R-4-tetA	plasmid	CP066257.1	*Klebsiella pneumoniae* strain CRKP78R	*Homo sapiens*	2018.05.23	China:Hangzhou
	p4_L39	plasmid	CP033957.1	*Klebsiella pneumoniae* strain L39_2	*Homo sapiens*	2018	China:Zhejiang
	pWP8-W19-CRE-01_3	plasmid	AP022271.1	*Raoultella ornithinolytica* strain WP8-W19-CRE-01	wastewater treatment plant effluent	2019.02.05	Japan:Tokyo
	p2481359-2	plasmid	CP067382.1	*Klebsiella grimontii* strain 2481359	*Homo sapiens*	2015.10	Switzerland
pS90-2.3_MDR1	pEF01	plasmid	CP040806.1	*Escherichia fergusonii* strain EFCF056	chicken	2017.05.04	China: Zhejiang
	pMV-u1-SK2-O-a	plasmid	CP085867.1	*Klebsiella pneumoniae* strain MV-u1-SK2-O	environmental swab veterinary clinic	2020	Switzerland
	p2018C01-046-3	plasmid	CP044371.1	*Klebsiella pneumoniae* strain 2018C01-046	*Homo sapiens*	2018	China: Taiwan

Cf.1	chromosome	CP085642.1	*Citrobacter freundii* strain Cf.1	bullfrog	2020.10.11	China: Guangxi Zhuang Nationality Autonomous Region
p24362-1	plasmid	CP051379.1	*Salmonella enterica* subsp. enterica serovar *Typhimurium* strain CVM 24362	swine	2002	USA:MO
